# Surface interactions and viability of coronaviruses

**DOI:** 10.1098/rsif.2020.0798

**Published:** 2021-01-06

**Authors:** Mehmet Onur Aydogdu, Esra Altun, Etelka Chung, Guogang Ren, Shervanthi Homer-Vanniasinkam, Biqiong Chen, Mohan Edirisinghe

**Affiliations:** 1 Department of Mechanical Engineering, University College London (UCL), Torrington Place, London WC1E 7JE, UK; 2 Science and Technology Research Institute, University of Hertfordshire, Hatfield AL10 9AB, UK; 3 School of Mechanical and Aerospace Engineering, Queen's University Belfast, Belfast BT7 1NN, UK

**Keywords:** COVID-19, SARS-CoV-2, surfaces, transmission, disinfectants

## Abstract

The recently emerged coronavirus pandemic (COVID-19) has become a worldwide threat affecting millions of people, causing respiratory system related problems that can end up with extremely serious consequences. As the infection rate rises significantly and this is followed by a dramatic increase in mortality, the whole world is struggling to accommodate change and is trying to adapt to new conditions. While a significant amount of effort is focused on developing a vaccine in order to make a game-changing anti-COVID-19 breakthrough, novel coronavirus (SARS-CoV-2) is also developing mutations rapidly as it transmits just like any other virus and there is always a substantial chance of the invented antibodies becoming ineffective as a function of time, thus failing to inhibit virus-to-cell binding efficiency as the spiked protein keeps evolving. Hence, controlling the transmission of the virus is crucial. Therefore, this review summarizes the viability of coronaviruses on inanimate surfaces under different conditions while addressing the current state of known chemical disinfectants for deactivation of the coronaviruses. The review attempts to bring together a wide spectrum of surface–virus–cleaning agent interactions to help identify material selection for inanimate surfaces that have frequent human contact and cleaning procedures for effective prevention of COVID-19 transmission.

## 1. Introduction

Having their first examples observed as zoonotic types of viruses back in the 1960s, the term coronavirus covers a broad range of respiratory virus family, which is responsible for various diseases, showing a variety of symptoms like mild and common cold while in certain situations severe respiratory syndromes can also be observed as a consequence of contact with that virus family [1,2]. Before the first significant threat emerged, there was only one type of coronavirus, called human coronavirus (human-CoV), that was known to infect humans and display common cold like symptoms as well as acute respiratory illnesses that would cause more serious problems [3]. Furthermore, until now, there were two types of coronaviruses which made a noticeable impact by showing aggressive symptoms on a level of epidemy, namely severe acute respiratory syndrome (SARS) caused by SARS-CoV back in 2002 and Middle East respiratory syndrome (MERS) originating from MERS-CoV in 2012 which are both directly linked to betacoronaviruses (β-CoV) that are known to be able to infect mammalians [4,5]. Coming from the same β-CoV genus, the most serious and recent variety of coronavirus (COVID-19) has emerged to cause a worldwide pandemic which was announced by the World Health Organization (WHO) on 11 March 2020 [6]. As of 20 September 2020, the weekly epidemiological update announced by the WHO showed that the current state of the epidemic has reached to 30 675 675 confirmed cases of which 954 417 were fatal meaning that the mortality rate was around 3.1% [7]. As the infection rate rise over time increases, it has become a severe threat worldwide on many levels, such as public health, the global economy and the social wellbeing of individuals. In order to overcome this pandemic and tackle its spread, requirements for understanding the spreading mechanism becomes essential. Latest reports have indicated that COVID-19 spreads through different ways and the most surprising discussion is made on the rare possibility of transmission via the digestive tract because of the successful isolation of the virus from faeces, which gives the situation a whole new perspective in terms of transmission [8,9]. However, among all rare possibilities, the principal transmission route of the virus is reported to be through the respiratory tract, using droplets and excretions originating from the respiratory system, spreading via coughing and direct contact of those vectors with the mucous membranes of humans [10]. This is also followed by airborne transmission since the smaller droplets tend to evaporate much faster compared to the bigger ones. Therefore, the virus continues to travel in the air, which creates a significant risk of transmission indoors and in crowded places such as supermarkets, theatres, offices and public transport [11]. Beside the direct contact made with the virus carrying droplets, there is a substantial risk of indirect contact with virus-contaminated surfaces that individuals interact on a daily basis. Nevertheless, the droplet and airborne transmission are reported to be one of the major ways for the virus to spread and usage of personal protective equipment is the mainly adopted way to prevent the spread of the COVID-19, eliminating the presence of the coronavirus from the surfaces that we interact on a daily basis. This is something that has to be investigated thoroughly and is equally important as the current precautions that are taken to slow down the spread of COVID-19 are frequently revised.

In order to remove the virus from those surfaces, the healthcare industry has already adopted various types of disinfectants and biocidal agents such as alcohol, hydrogen peroxide and sodium hypochlorite [12]. However, the importance of a detailed investigation about coronavirus persistence on inanimate surfaces and removal of the virus from those surfaces is a rather undeniable truth than a topic of debate! Therefore, this review focuses on surfaces that pose a risk to became vectors in terms of transmitting coronavirus, how long the virus can survive on different surfaces and interaction of the coronavirus and different material types as well as cleaning agents for optimized removal of the coronaviruses. It also elucidates how metallic nanoparticles, antiviral drugs and nanotechnological approaches can be used to reduce the transmission rate of infection and prevent any future outbreaks.

## 2. Transmission of COVID-19

**Figure 1.**
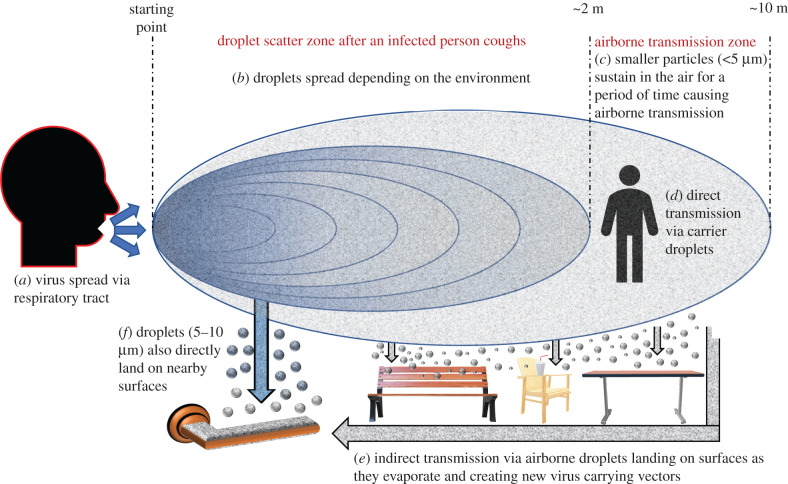
Estimated route and role of the respiratory droplets in terms of spreading COVID-19 after an infected person coughs.

Respiratory droplets, which are bigger than 5–10 µm in diameter and droplet nuclei that are smaller than 5 µm in diameter are the primary reasons for respiratory infections to be transmitted from one to another [13]. It has been previously reported that droplets which are bigger than 5 µm are most likely to be encountered via the upper respiratory tract such as nose, throat and oropharynx, respectively, while the smaller droplet nuclei can pass through and deposit inside the lower respiratory system elements such as bronchi and alveoli when they are inhaled [14]. It is certain that there is a significant risk of being infected by coronavirus after being exposed to those respiratory droplets and droplet nuclei coming from an infected person who has respiratory symptoms, especially when the virus carrying droplets contacts with the mucous membrane of the mouth, the nose as well as conjunctiva of eyes [15,16]. Furthermore, there is another direct risk factor called airborne transmission, where the air becomes a vector to carry and also hold the droplet nuclei, thus small particles carrying the virus can be inhaled directly to the lower respiratory tract. Figure 1 summarizes the transmission of COVID-19 by schematizing the spread of the droplets and droplet nuclei after coughing. It has been previously reported that the virus can survive up to 3 h as droplets in the air, after being coughed out by the infected person [17]. That kind of a mechanism poses a significant threat for indoor and crowded environments in both a direct and indirect manner where the droplet nuclei can carry the virus during an indoor scenario allowing individuals to have direct contact with the virus [18].

This can be followed by another mechanism of transmission which is more uncertain and elusive as the droplet nuclei spreads through an ‘unpredictable’ route, travels in the air up to tens of metres as their liquid content evaporates, eventually precipitating on the surfaces of indoor elements such as door handles, handrails, or else where there is frequent human interaction, thus making inanimate objects vectors of transmission [19]. Furthermore, β-CoV genus coronaviruses such as SARS-CoV, MERS-CoV and SARS-CoV-2 are reported to have heterogeneity in terms of transmissibility, which defines an extremely aggressive spreading phenomenon particularly observed in hospital environments [20]. Nowadays, various types of personal protective equipment (PPE) have been widely adopted by hospital staff and individuals in daily life in order to tackle this aggressive transmission [21]. Especially, usage of different types of face masks and making a habit of wearing PPE has successfully provided a proper way of protection to the wearers against such risks [22,23]. However, contact routes created by inanimate objects acting as vectors of transmission after being exposed to the respiratory tracts of an infected person still carries a significant risk in terms of transmission since they are not easy to track. Therefore, a detailed investigation of the contact routes and transmission via those contact surfaces plays a key role in controlling the outbreak by better management of the risky surfaces and regulation of those routes in terms of hygiene.

## 3. Taxonomy, action mechanism and receptor recognition of coronaviruses

**Figure 2.**
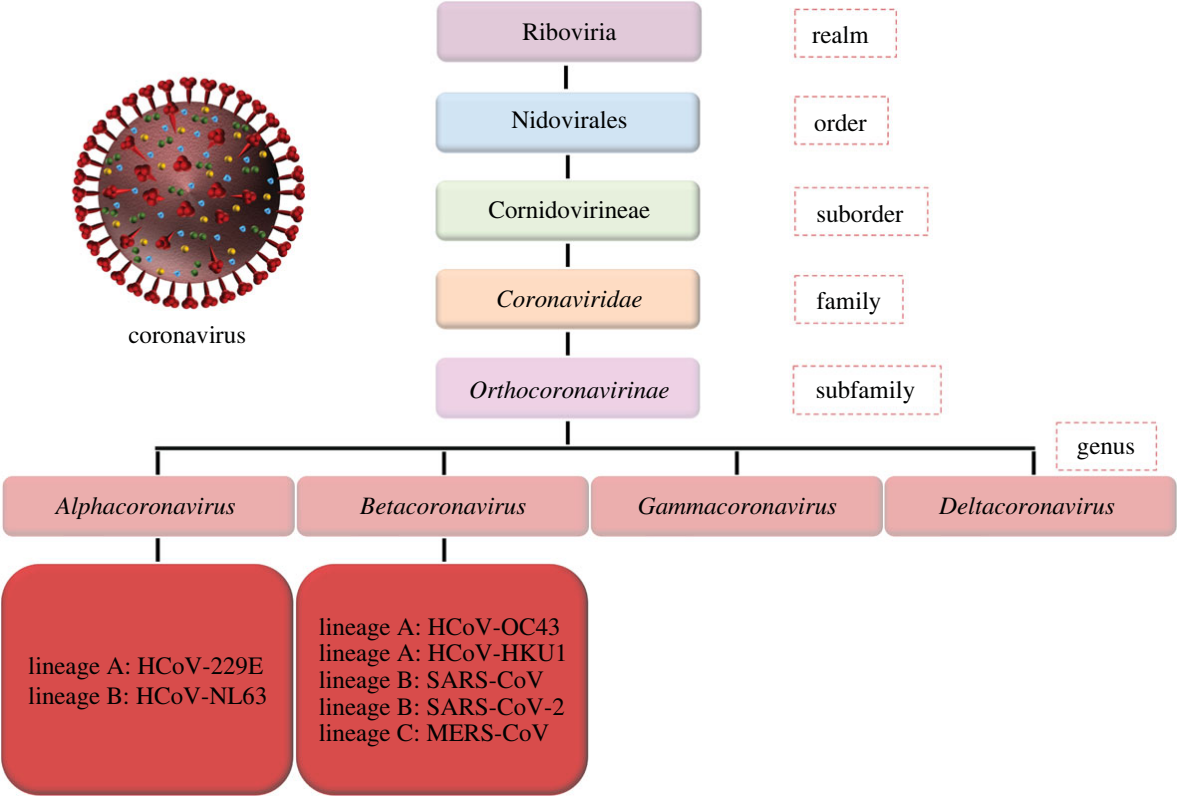
Taxonomy of the SARS-CoV-2 and its close relatives. Adapted from Tang *et al*. [24]. Under a CC BY License, Copyright 2020, PLOS.

Coming from the *Coronaviridae* family (figure 2), SARS-CoV-2 is a member of β-CoV genus and reported to be similar to the SARS-CoV, MERS-CoV and other human coronaviruses in terms of taxonomy and genetics [24,25]. Therefore, it must be accurate and beneficial for scientists to take those ‘relatives’ of SARS-CoV-2 into account since there are very limited data about this newly emerged threat and prevention measures adopted during previous research and virus outbreaks play a significant role while developing new strategies against SARS-CoV-2. Thus, coronaviruses are known to carry positive-stranded and encapsulated RNA that is meant to be delivered into the host cells in order to start the infection. Cell to virus interactions via proteins on each surface plays a crucial role in this matter, determining the host range of the coronaviruses [26]. Moreover, understanding the structure of the coronaviruses would be helpful in the future for solving the attachment mechanism to the inanimate surfaces as well.

**Figure 3.**
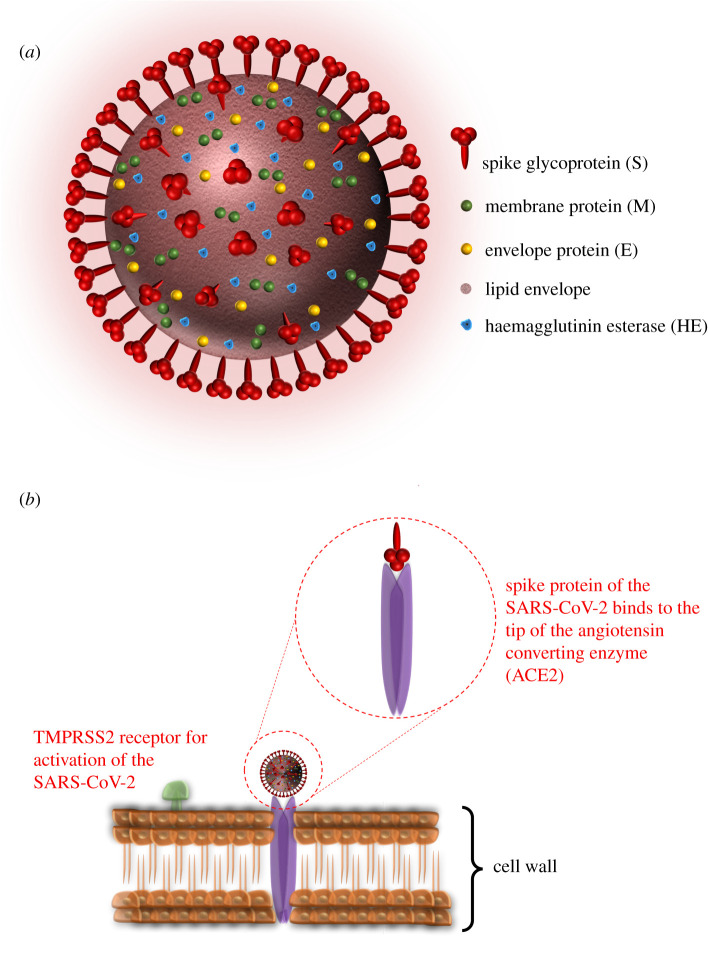
Schematic design of (*a*) shape and proteins of the coronavirus and (*b*) cell entry mechanism via spike proteins.

Responsible for the current pandemic, the SARS-CoV-2 coronavirus carries a different structure of proteins which are membrane glycoprotein (M), spike protein (S), haemagglutinin esterase (HE) and envelope (E) protein as shown in figure 3*a* and the nucleocapsid protein (N) can be found inside the lipid layer, which accompanies the viral RNA and protects it. Even though the role of those proteins is not fully understood, recent studies showing that M is the most frequently detected protein on the virus surface, which is responsible for the shape of the membrane of the coronavirus and is also believed to be included in the mechanism of the virus to cell binding interactions [27]. On the contrary, E is found in small quantitates and responsible for the release of the viral content. It has been previously reported that ion channel activity on this protein is required for the development of the disease in terms of pathological activity [28]. Finally, it would be hard to overstate the importance of S and HE on the cell binding mechanism of the coronaviruses. S proteins which are reported to be a class I fusion protein are responsible for attachment to the host receptors [29,30]. In a scenario where a coronavirus interacts with a human cell, identification of the cell is made via the enzymes on it. After the S protein of the SARS-CoV-2 is primed by the TMPRSS2 enzyme on the cell wall, engagement on the attachment receptor angiotensin-converting enzyme 2 (ACE2) of the cell is made by S proteins of the coronavirus in order to initiate the entrance procedure on the inside of the host cell (figure 3*b*) [31]. As another important aspect in cell entry mechanism, HE protein is also believed to play a key role to enhance and support the functions of S as well as in allowing the virus to transmit through the mucosal tissue [32].

## 4. Persistence of the coronaviruses on different surfaces

As one of the most important concepts in indirect transmission of the COVID-19, material type of the surfaces plays an important role since elements of inanimate environment could act as fomites (inanimate objects which can carry microbes, acting as vectors that can help spread of the viruses), allowing the virus to remain viable over a long timescale [33]. Healthy individuals contacting contaminated surfaces as part of their daily routine allow transmission to happen from an infected to a healthy person even though there is no direct contact. Hence, this elusiveness forces medical personnel and scientists to take serious precautions and reconsider the material selection on the most popular elements of door handles, handrails, or elevator buttons. In addition, researchers have reported studies evaluating the substantiality of different types of coronaviruses with different inoculums on different surfaces.

For the SARS coronavirus, Duan *et al*. [34] published the different strains of SARS coronavirus and evaluated the survival of the coronavirus on different surfaces. Results indicated that survival time of coronavirus with 10^5^ viral titre was 96 h on wood and glass, 96 h to 120 h on paper and 120 days on metal. A previous study by Lai *et al*. [35] has shown that in a disposable gown under varying inoculum values, coronavirus was found to be alive up to 48 h in 10^6^ viral titre which diminished to 24 h in 10^5^ and to 1 h in 10^4^ inoculum. Furthermore, the viability of SARS coronavirus on plastic surfaces has been reported to be up to 5 days in another study [36].

In terms of human endemic coronavirus, a previous study by Sizun *et al*. [37] demonstrated that under 5 × 10^3^ viral titre aluminium surfaces allowed coronavirus to stay alive for 2–8 h while on latex surgical gloves it was up to 8 h. On the other hand, Warnes *et al*. [38] reported a comprehensive analysis (10^3^ infectious titre) about how different materials allow coronavirus to stay infectious on them. Results indicated that steel, stainless steel, glass, silicone rubber, PVC, ceramic and teflon all allowed coronavirus to stay active for 5 days.

Previous work published by van Doremalen *et al*. [39] also revealed the viability characteristics of the MERS coronavirus on surfaces. After testing on steel and plastic under 10^5^ viral titre, results indicated the survival time of the MERS coronavirus was 48 h at 20°C and 8–24 h at 30°C on both surfaces.

**Figure 4.**
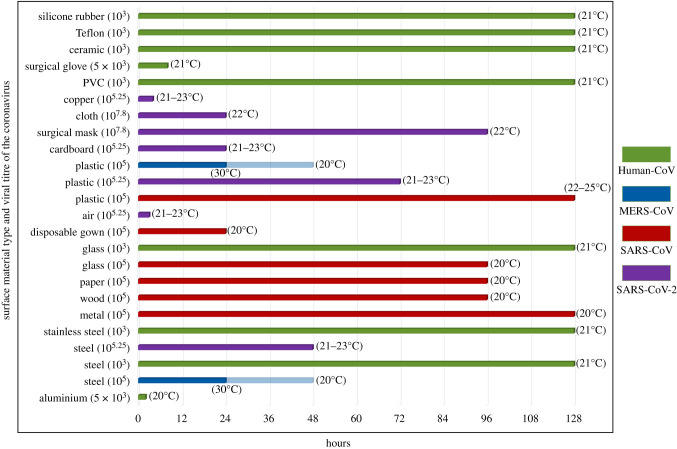
Persistence and viability of different coronavirus strains with different viral titres on most commonly used surface material types (human-CoV: human endemic coronavirus, MERS-CoV: Middle East respiratory syndrome, SARS-CoV: severe acute respiratory syndrome, SARS-CoV-2: COVID-19). (Data obtained from Duan *et al*. [34], Lai *et al*. [35], Chan *et al*. [36], Sizun *et al*. [37], Warnes *et al.* [38], van Doramalen *et al*. [17,39], Chin *et al*. [40].)

There are limited amount of studies published about the survival characteristics of SARS-CoV-2 on different surfaces. van Doremalen *et al*. [17] have recently reported about surface stability of SARS-CoV-2 and comparison of those characteristics with SARS-CoV. At 21 to 23°C degrees of ambient temperature with 10^5.25^ viral titre, steel, air, cardboard and copper were tested, and results indicated that the SARS-CoV-2 was able to survive 3 h in air, 4 h on copper, 24 h on cardboard, 48 h on steel and more than 72 h on plastic. In addition, Chin *et al*. [40] reported that persistence of the SARS-CoV-2 was 96 h on surgical masks and 24 h on cloth under 10^7.8^ viral titre at 22°C. In figure 4, various surfaces and survival of different types of coronaviruses under different conditions is collated by the authors' of this review.

## 5. SARS-CoV-2 adsorption mechanism on different inanimate surfaces

It has recently been found that surface contamination is very important in terms of transmission of SARS-CoV-2 [41]. Continuous recontamination with contaminated environmental surfaces transfers the infectious virus between humans. Evaluation of the adhesion mechanisms of SARS-CoV-2 on different inanimate surfaces is crucial for preventing deposition and designing removal methods. The physico-chemical adherence and the persistence of SARS-CoV-2 differ with the characteristics of the inanimate surfaces and the virus outer surface proteins, as well as on the surrounding environmental conditions, such as air temperature, relative humidity (RH) and sunlight [42–44].

The adhesion mechanism of SARS-CoV-2 on environmental surfaces has yet to be adequately delineated, but it has been predicted that it is primarily driven by electrostatic attractions (e.g. pH, isoelectric point (pI) and ionic strength), then hydrophobic effects, and minorly non-covalent bonds (e.g. van der Waals forces) which could all govern the binding of the S protein to solid surfaces [45].

Since the surface charge of viruses differs with the varying pH, disruption of electrostatic interactions between viruses and inanimate surfaces usually involves altering the pH, pI and ionic strength to manipulate the persistence of the virus [46,47]. The virus is exceptionally stable over a wide pH range (3–10) while showing low stability at pH values (3–5) compared to alkaline pHs (9–12) [48]. The pI of SARS-CoV-2 has not been defined to understand the adhesion mechanism of the virus to inanimate surfaces, but it is assumed that they are largely affected by the isoelectric properties of the surface glycoproteins (M and N proteins). Moreover, reduction in the ionic strength of the surrounding medium between viruses and inanimate surface results in increased electrostatic interactions and reduce the surface aggregation of adhered viruses [49]. Van der Waals forces also play a minor role in the physical adsorption in the short distance between the virus and inanimate surfaces [50].

The E protein is a highly hydrophobic lipid layer of SARS-CoV-2 shielding the whole virus and altering the hydrophobicity of the surface can inhibit the adherence of the virus to surfaces while inactivating this protein [51]. With hydrophobic effects, adhesion can be minimized in the interfacial area between water and apolar surfaces on the viruses, thereby reducing the binding by decreasing the apolarity of the virus [52].

Compared to indoor conditions (20–24°C, 40–50% RH), the stability of SARS-CoV-2 drastically reduces (greater than 3 log_10_) at a temperature above 38°C and RH levels higher than 95% and it causes weaker adhesion for survivor viruses to solid inanimate surfaces [48]. Additionally, natural sunlight (low level of UV irradiance 250–280 nm) can be used on the inactivation of the virus on surfaces with an almost 1000-fold reduction in viral infectivity [53].

Survival of the virus on non-porous material surfaces (e.g. stainless steel, plastic, latex and glass) was found to be higher than those on porous material surfaces (e.g. paper and cotton). It has been found that these porous surfaces can capture viruses in their matrix and also dehumidifies viruses while accelerating the destruction process of envelopes and thus making the virus less infectious [54]. Also, topographic irregularities, texture and roughness of an inanimate surface play a role on virus deposition [55]. Decreased roughness of a surface and creation of a micro-/nano-multiscale textured surface can reduce the contact surface area available for virus adhesion and decrease surface stability for SARS-CoV-2 [56].

The findings on SARS-CoV-2 survival on different inanimate surfaces are still not sufficient to describe the mechanism by which this virus adheres to inanimate surfaces and further epidemiological research is needed on this topic.

## 6. Inactivation and removal of the coronaviruses

### 6.1. Surface disinfectant agents

Inactivation and removal of the coronaviruses from surfaces is an important topic to prevent the spread of the virus and it requires the incorporation of different chemicals depending on the type of the surface. Since the physical contact between inanimate surfaces and hands are frequently occurring during a regular day, cleaning agents are being investigated for their antiviral effects. In order to deactivate coronaviruses, by reducing and or hindering their ability to cause infections, suspension tests in which the virus meets the disinfectant in a suspension has been widely adopted along with the carrier tests where the virus and disinfectant come together on a certain surface.

During suspension tests, each chemical was diluted by volume before the application. Ethanol was frequently used in increasing concentrations such as 78%, 80%, 85% and 95% with 30 s of exposure time on different coronavirus types. Reduction of the viral infectivity was reported between 4.3 to 5.5 log_10_ [57–59]. In the same study reported by Rabenau *et al*. [57] formaldehyde was also used in 0.7% and 1% ratios under 2 min of exposure and viral infectivity reduction was at least 3 log_10_. Just like ethanol, povidone iodine was also investigated under different concentrations as well as against different types of coronaviruses from the beta family. Results indicated that the usage of povidone iodine in various ratios ranging from 0.23% to 7.5% under a designated exposure time of 15 s ended up with 4.4 to 5 log_10_ reduction in viral titre [60,61]. On the other hand, 2-propanol is another cleaning agent showing promising results. Results of testing against different types of coronaviruses indicated that the 70%, 75% and 100% ratios of 2-propanol resulted in 3.3 to 4.0 log_10_ viral infectivity reduction under 30 s of exposure [57,58]. It was also reported that 0.5% hydrogen peroxide with 1 min of exposure time reached the same viral infection reduction ratio observed in 0.5% glutardialdehyde with 2 min of exposure time, having resulted in at least 4 log_10_ decrease of the viral infectivity in both cases [57,62]. There are other cleaning agents proven to be ineffective against coronaviruses. Even though the exposure time was extended to 10 min, 0.2% benzalkonium chloride reported no reduction of viral infectivity [63]. Moreover, incorporation of two different cleaning agents was also tested in order to reduce the viral infectivity of the coronaviruses. Using a blend of 45% 2-propanol and 30% 1-propanol, viral infection reduction was found to be at least 4.3 log_10_ under 30 s of exposure, showing a slight increase compared to using 2-propanol only [59].

**Figure 5.**
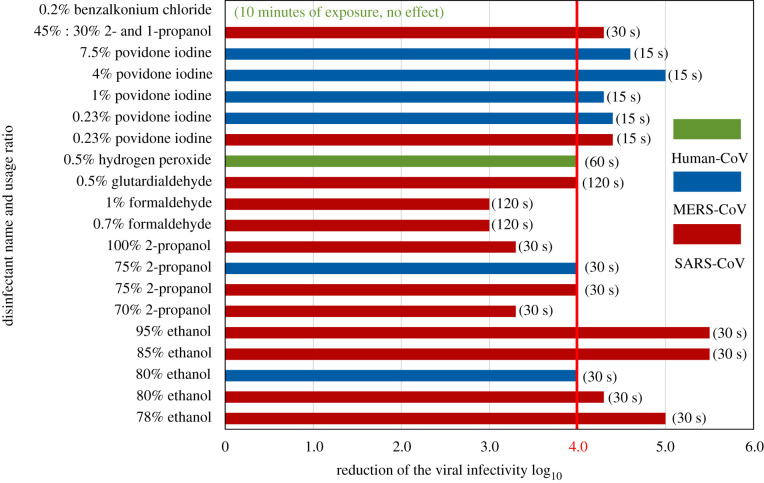
Minimum reduction of viral infectivity values obtained in suspension tests using various disinfectants in different concentrations against different coronaviruses (human-CoV: human endemic coronavirus, MERS-CoV: Middle East respiratory syndrome, SARS-CoV: severe acute respiratory syndrome, SARS-CoV-2: COVID-19). (Data obtained from Rabenau *et al*. [57,59], Siddharta *et al*. [58], Eggers *et al*. [60,61], Omidbakhsh *et al*. [62], Wood *et al*. [63].)

Figure 5 demonstrates the minimum viral infectivity reduction each disinfectant can provide under different concentrations against different types of coronaviruses with the given exposure time. According to European standards efficiency criteria, in order to be qualified as an antiseptic and provide antiviral features, reduction of the viral infectivity must be at least 4 log_10_ [64]. Therefore, according to suspension test results, ethanol, glutardialdehyde and povidone iodine qualify as proper cleaning agents in order to deactivate the coronavirus on various surfaces since they were able to meet the 4 log_10_ threshold and go beyond that value. It is also noteworthy that 70% ethanol is also the recommendation of the WHO [65]. In parallel with the findings, the rest of the materials can also provide the same level of reduction in terms of viral infectivity with some of the ratios tested except benzalkonium chloride and formaldehyde, since both of these stayed well below the given threshold.

**Figure 6.**
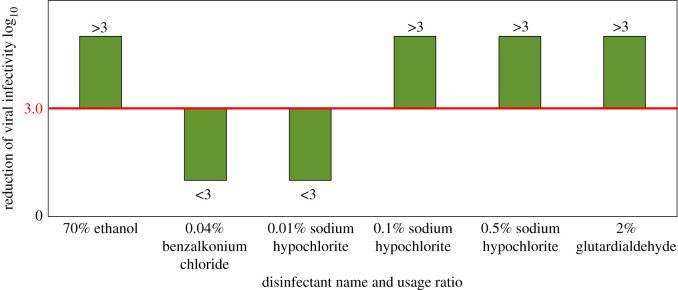
Comparison of results of the carrier tests in terms of selected threshold 3 log_10_ using different disinfectants against human-COV on a stainless-steel surface for 1 min of exposure at room temperature (22–24°C). (Data obtained from Sattar *et al*. [66].)

Regardless of the suspension tests, the carrier test of the different cleaning agents was introduced by Sattar *et al.* [66]. Under exactly 1 min of exposure time on a stainless-steel surface at room temperature (22–24°C), 70% ethanol, 2% glutaraldehyde, 0.01, 0.1 and 0.5% sodium hypochlorite and 0.04% benzalkonium chloride diluted by volume were individually investigated against coronavirus and reduction of the viral infectivity was assessed according to a selected threshold of 3 log_10_ viral infectivity reduction. The main expectation of viral infectivity reduction on hard objects was reported to be 2 to 4 log_10_ [67]. As can be seen in figure 6, results indicated that the ethanol, sodium hypochlorite in 0.1% and 0.5% ratios, and glutaraldehyde falls above the 3 log_10_ threshold and can be considered as effective and promising cleaning agents for coronaviruses. On the other hand, viral infectivity reductions of the benzalkonium chloride and 0.01% sodium hypochlorite were observed to be under the selected threshold and falls slightly behind the other disinfectants in terms of viral infection reduction ability [66].

Even though these disinfectant solutions would sound promising for inanimate surfaces, our hands are more delicate and constant usage of chemicals such as alcohol will also damage the lipid structure of the skin and compromise the integrity of it, allowing it to be more exposed and vulnerable to the microbes. Therefore, usage of the surfactants such as soap in order to remove coronavirus from hands can be more realistic, cost effective and beneficial.

### 6.2. Antiviral nanoparticles

**Figure 7.**
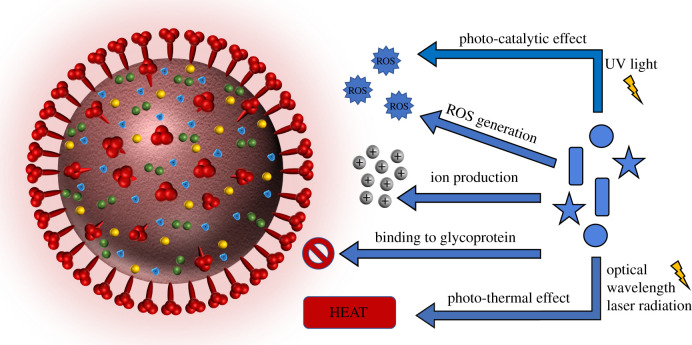
Schematic figure showing possible mechanisms of action of nanoparticles against enveloped viruses.

A range of nanotechnological concepts, including the use of silver nanoparticles, have shown to exhibit antiviral activity against a broad range of viruses with similar mechanisms of action to antiviral drugs and their performance against microbial cells [68,69]. As schematically illustrated in figure 7, it has been found that some nanomaterials can induce antiviral activity through the production of ions, generation of reactive oxygen species (ROS), photothermal and photocatalytic effects, and the interaction with viral glycoproteins to inhibit their binding and penetration [69]. Some of the nanoparticles are known to release ions in suspension and certain ions have shown antiviral activity against coronavirus. Metal ions can interact with essential viral enzymes but other undefined mechanisms also prevail [70–72]. For example, Zn^2+^ ions were able to inhibit SARS-CoV replication through the inhibition of RNA-dependent RNA polymerase elongation [71]. Similarly, Warnes *et al*. [38] reported that copper surfaces were able to destroy the envelope and surface spikes of human coronavirus 229E, resulting in altered morphology, and therefore expose viral genome which was also destroyed. Ions produced by the copper surface was responsible for the inactivation of the coronavirus, while the generation of ROS on the copper surface enhanced the antiviral activity. Although bulk material was tested in this investigation, studies have shown that the release of ions from metals is proportional to the surface area, and the exposure of nanoparticles can increase ROS generation. Thus, copper nanoparticles may exhibit increased antiviral activity compared to bulk copper [72,73] and some masks are doped with copper nanoparticles and use this property already.

While the interaction with coronavirus glycoproteins has not been reported, the antiviral activity of metal nanoparticles against other viruses has led to hypothetical theories. Since gold, silver and silver–copper nanoparticles have a proven ability to bind to HIV glycoprotein and inactivate the viral binding and penetration into host cells, it is worth further exploring for similar mechanisms against coronavirus [74–77]. Additionally, it is also worth mentioning the potential of photothermal and photo-catalytic properties of metallic nanoparticles to disinfect surfaces. It has been reported that coronaviruses can be inactivated by heat with the temperature dependent on exposure time; overall 30 min exposure at 60°C can reduce coronavirus by at least 4 log_10_, while 1 min at 80°C has the same reduction rate [78]. Currently, photothermal therapy is used to inactivate cancer cells and has also shown to inactive virus cells [79]. Although it has not been tested against coronaviruses, this approach is possible with other viruses. As an example, murine leukaemia virus has been successfully inactivated using gold nanorods excited by 805 nm laser [80]. In parallel, photocatalytic effect occurs when ROS is produced from the excitement caused by UV light exposure [79]. Nakano *et al*. [81] have reported influenza virus inactivation through the photocatalytic activity of titanium dioxide nanoparticles. As discussed above, the generation of ROS can induce antiviral activity towards coronavirus, therefore nanoparticles with photo-catalytic properties is another possible approach towards inactivation of coronaviruses.

The use of ‘new’ materials like graphene and its derivatives in combating coronavirus is yet to be fully investigated. However, these materials can be very effective against viruses since they display certain antiviral mechanisms such as production of ROS, having negative charge, showing physio-chemical interactions with viruses, competitive inhibition mechanism and inactivating enveloped RNA viruses [82–84]. Additionally, action mechanisms of graphene and valuable features of its derivatives have already inspired researchers for adopting these interactions to propose new studies against SARS-CoV-2 in different application areas and roles [85].

### 6.3. Role of antiviral drugs

Another concept that can inactivate coronaviruses is antiviral drugs. Even though it is not directly related to inanimate surfaces, understanding the virus inactivation mechanism of antiviral drugs and their relationship to proteins of the SARS-CoV-2 might also be beneficial for further understanding of possible coating materials which can be used on inanimate surfaces in terms of replicating the same effect. Despite years of intensive research on antiviral drugs against human coronavirus, there is currently no approved or effective treatment for SARS-CoV, SARS-CoV-2 or MERS-CoV. However, there are ongoing investigations on suggested FDA approved drugs or repurposed drugs as antiviral candidates [86,87].

Repurposed drugs have shown successful results that target the viruses using a variety of predicted mechanisms, although they are not currently recommended for use outside clinical trials [86,88,89]. As a whole, antiviral drugs exhibit different mechanisms of action such as binding and fusion inhibitors, enzyme and channel blockers as well as polymerase, kinase, protease and cyclophilin inhibitors; each targeting particular stages of the viral cycle. The interaction of the antiviral drugs and S proteins of the coronaviruses might be the most beneficial to focus on since it can give perspective to understand how other materials might interact with those spike proteins. As the viral cells attach to host cells through interactions between surface proteins and receptors, they can subsequently penetrate into host cells [90]. Certain drugs can target and inhibit the binding of viral cells to host cells or inhibit fusion, for example inhibiting viral surface proteins [91]. Examples of repurposed drugs that have interactions with spike and surface proteins are shown in table 1 and a common mechanism of actions include blocked entry (which targets the attachment and penetration of the virus into the host) [88].

**Table 1.** Examples of repurposed drugs that have shown positive activity against coronavirus in terms of blocking cell entrance by inhibiting the virus and cell interactions.

drug	dosage	target virus	approved clinical status	main result	predicted antiviral mechanism of action	reference
Camostat	30 mg kg^−1^	MERS-CoV SARS-CoV	chronic pancreatitis	survival rate of approximately 60%	blocked entry	[92]
K11777	0.68–46.12 nM 0.35–1.04 µM	MERS-CoV SARS-CoV	Chagas disease	50% infectivity inhibition^(IC50)^ 90% viral yield reduction^(IC90)^	blocked entry	[92]
toremifene	60 mg day^−1^ for 2 weeks	SARS-CoV-2 model	cancer	blood concentration of the drug reached three times over the required IC50	blocked entry via glycoprotein inhibition	[93]
emodin	50 µl	SARS-CoV-2 protein- pseudo-typed retrovirus	cancer	inhibition of the S protein-pseudo-typed infectivity by 94.12 ± 5.90%	blocked entry via inhibition of virus protein and host receptor binding	[94]
ivermectin	5 µM/48 h	SARS-CoV-2	anti-parasitic agent	99.98% reduction in viral RNA	inhibiting nuclear import of viral protein	[95]
chloroquine	—	SARS-CoV-2 model	malaria	virus–cell attachments revealed in detail relating to molecular properties of used drugs	attachment inhibition	[96]
hydroxychloroquine	—					

### 6.4. Nanotechnological approaches against COVID-19

Nanotechnology is a highly complex but promising concept that has already been widely adapted against COVID-19 since the SARS-CoV-2 virus and nanotechnological tools operate on a similar size scale [97]. Understanding, altering and controlling those interactions can help researchers to develop rapid and more accurate virus detection implementations and better control tools as well as more effective PPE [98–100].

Along with the antiviral properties of the nano-sized particles discussed in the previous sections, nanotechnology can also be integrated into this ongoing COVID-19 battle in terms of detection of the disease. The currently employed main diagnosis method is based on the nucleic acid testing mechanism namely reverse transcription-polymerase chain reaction (RT-PCR). However, this method comes with certain limitations in terms of real-life applicability since the RT-PCR test may be incapable of revealing patients that are not showing symptoms. Additionally, not every healthcare centre or hospital, especially smaller organizations without enough facilities, can handle the massive workload caused by increased demand due to insufficient PCR testing capabilities and, finally, number of kits and reagents available are insufficient to meet spiking demand in general [101]. With the aim of compensating for these problems, a former biosensor-based pathogenic detection system has been incorporated with the current reverse transcription (RT) method by Zhu *et al*. [102], resulting in a one-step, effective and low-cost diagnosis tool for SARS-CoV-2 suitable for laboratories and healthcare centres that are resource limited. As reported by Chen *et al*. [103], a novel way of virus detection can also be achieved by using biomimetic nanoparticles interacting with viruses for enhancing their exposure to certain detection tools. Therefore, it can be said that nanotechnological approaches hold remarkable potential in terms of improving testing and detection capabilities. Point of care testing concept was also built on strong fundamentals owing to nanotechnology developments. As stated by Udugama *et al*. [99], previous studies showing that gold nanoparticles can help develop new and enhanced detection methods that can be suitable for point of care applications without needing a laboratory to send samples to and fro can be extremely beneficial in certain scenarios. Furthermore, nanotechnology can also create a difference when it comes to controlling the spread of the disease. Medhi *et al*. [104] demonstrated how nanotechnology-based strategies can be beneficial for blocking cell attachment and controlling the spread of the virus. Not only limited to that, a recent study proposed usage of nanoparticles to create decoy targets for SARS-CoV-2 to attach, absorbing viruses and hindering their chance to contact cells to reduce the rate of developing an infection [105]. Moreover, previous studies reveal how nanotechnological approaches can reach out further by pioneering the creation of improved PPE and nano-sized drug delivery systems to overcome COVID-19 [106,107].

Among mentioned prevention methods which are the first line of defence for tackling the overwhelming course of the pandemic, the real challenge lies in vaccine development studies. The importance of nanotechnological approaches has already been suggested in terms of developing a safe and effective vaccine against COVID-19 [108–110].

## 7. Conclusion and future perspectives

It is a fact that the advent of the pandemic has changed the tide of our lives and as human beings we are all obliged to understand and adapt new aspects in order to protect ourselves and society. SARS-CoV-2, the coronavirus strain which is responsible for current COVID-19 pandemic has already spread all over the world and keeps transmitting between individuals, targeting vulnerable people more, as well as the healthcare industry and worldwide economy. As the world struggles to find a vaccine or an effective drug in order to overcome this threat as a whole, these goals are still quite far away from reality as we have so much to develop with respect to these concepts. Therefore, this review focuses on viability of the coronaviruses on inanimate surfaces since they are crucial and frequent vectors of transmission as well as this knowledge sheds light on the disinfectant chemicals reported in previous studies in order to inhibit the infection ability of the coronaviruses. This can be done by analysing the morphology of the coronavirus, virus protein to cell enzyme interactions while considering the lessons learnt from the past such as the SARS epidemic and the MERS outbreak by comparing related coronavirus strains causing those diseases. Not only limited to that, mechanisms of virus response against surfaces, nanoparticles and antiviral drugs were also investigated from previously published articles. However, further epidemiological research is crucial and urgently needed on this topic to understand the behaviour of SARS-CoV-2 to come up with stronger protocols to fight against COVID-19.

## Data accessibility

This article has no additional data.

## References

[1] Suman R, Javaid M, Haleem A, Vaishya R, Bahl S, Nandan D 2020 Sustainability of coronavirus on different surfaces. J. Clin. Exp. Hepatol. 10, 386–390. (10.1016/j.jceh.2020.04.020) 32377058 PMC7201236

[2] Chen N et al 2020 Epidemiological and clinical characteristics of 99 cases of 2019 novel coronavirus pneumonia in Wuhan, China: a descriptive study. Lancet 395, 507–513. (10.1016/S0140-6736(20)30211-7) 32007143 PMC7135076

[3] Giwa AL, Desai A, Duca A 2020 Novel coronavirus COVID-19: an overview for emergency clinicians. Emerg. Med. Pract. 22, 1–21. 32105049

[4] European Centre for Disease Prevention and Control. 2015 Severe acute respiratory syndrome (SARS). Annual Epidemiological report for 2015 See https://www.ecdc.europa.eu/sites/portal/files/documents/AER_for_2015-SARS.pdf

[5] Yang D, Leibowitz JL 2015 The structure and functions of coronavirus genomic 3′ and 5 ends. Virus Res. 206, 120–133. (10.1016/j.virusres.2015.02.025) 25736566 PMC4476908

[6] WHO Coronavirus disease. 2019 2020 (COVID-19) situation report – 76. Geneva: WHO; 5 April 2020 See https://www.who.int/docs/default-source/coronaviruses/situation-reports/20200405-sitrep-76-covid-19

[7] WHO Coronavirus disease. 2019 (COVID-19). 2020 Weekly Epidemiological Update 20 September 2020 See https://www.who.int/docs/default-source/coronaviruse/situation-reports/20200921-weekly-epi-update-6.pdf?sfvrsn=d9cf9496_6.

[8] Zhang H et al 2020 The digestive system is a potential route of 2019-nCov infection: a bioinformatics analysis based on single-cell transcriptomes. Gut 69, 1010–1018. (10.1136/gutjnl-2020-320953)

[9] Zang W et al 2020 Molecular and serological investigation of 2019-nCoV infected patients: implication of multiple shedding routes. Emerg. Microbes. Infect. 9, 386–389. (10.1080/22221751.2020.1729071) 32065057 PMC7048229

[10] Li Q et al 2020 Early transmission dynamics in Wuhan, China, of novel coronavirus-infected pneumonia. N. Engl J. Med. 382, 1199–1207. (10.1056/NEJMoa2001316) 31995857 PMC7121484

[11] Wilson NM, Norton A, Young FP, Collins DW 2020 Airborne transmission of serve acute respiratory syndrome coronavirus-2 to healthcare workers: a narrative review. Anaesthesia 75, 1086–1095. (10.1111/anae.15093) 32311771 PMC7264768

[12] Kampf G 2018 Antiseptic stewardship: biocide resistance and clinical implications. Cham, Switzerland: Springer International Publishing.

[13] World Health Organization. 2014 Infection prevention and control of epidemic- and pandemic-prone acute respiratory infections in health care See http://apps.who.int/iris/bitstream/handle/10665/112656/9789241507134_eng.pdf.

[14] Atkinson J, Chartier Y, Pessoa-Silva CL, Jensen P, Li Y, Seto WH 2009 Natural ventilation for infection control in health-care settings. Geneva, Switzerland: World Health Organization. 23762969

[15] Otter JA, Donskey C, Yezli S, Douthwaite S, Goldenberg SD, Weber DJ 2016 Transmission of SARS and MERS coronaviruses and influenza virus in healthcare settings: the possible role of dry surface contamination. J. Hosp. Infect. 92, 235–250. (10.1016/j.jhin.2015.08.027) 26597631 PMC7114921

[16] Dowell SF et al. 2004 Severe acute respiratory syndrome coronavirus on hospital surfaces. Clin. Infect. Dis. 39, 652–657. (10.1086/422652) 15356778 PMC7107915

[17] van Doremalen N et al 2020 Aerosol and surface stability of SARS-CoV-2 as compared with SARS-CoV-1. N. Engl. J. Med. 382, 1564–1567. (10.1056/NEJMc2004973) 32182409 PMC7121658

[18] Morawska L, Cao J 2020 Airborne transmission of SARS-CoV-2: the world should face the reality. Environ. Int. 139, 105730 (10.1016/j.envint.2020.105730) 32294574 PMC7151430

[19] Morawska L, Johnson G, Ristovski Z, Hargreaves M, Mengersen K, Corbett S, Chao CYH, Li Y, Katoshevski D 2009 Size distribution and sites of origin of droplets expelled from the human respiratory tract during expiratory activities. J. Aerosol. Sci. 40, 256–269. (10.1016/j.jaerosci.2008.11.002) PMC7126899 32287373

[20] Wong G, Liu W, Liu Y, Zhou B, Bi Y, Gao GF 2015 MERS, SARS, and Ebola: the role of super-spreaders in infectious disease. Cell. Host Microbe. 18, 398–401. (10.1016/j.chom.2015.09.013) 26468744 PMC7128246

[21] Public Health England. 2020 Considerations for acute personal protective equipment (PPE) shortages See https://www.gov.uk/government/publications/wuhan-novel-coronavirus-infection-prevention-and-control/managing-shortages-in-personal-protective-equipment-ppe.

[22] Ahmed J, Harker A, Edirisinghe M 2020 Covid-19: facemasks, healthcare policies and risk factors in the crucial initial months of a global pandemic. Med. Devices Sens. (10.1002/mds3.10120)

[23] Alenezi H, Cam M, Edirisinghe M 2020 A novel reusable anti COVID-19 transparent face respirator with optimized airflow. Bio-Des. Manuf. (10.1007/s42242-020-00097-1) PMC7520078 33014512

[24] Tang D, Comish P, Kang R 2020 The hallmarks of COVID-19 disease. PLoS Pathog. 16, e1008536 (10.1371/journal.ppat.1008536) 32442210 PMC7244094

[25] Rabaan AA et al. 2020 SARS-CoV-2, SARS-CoV, and MERS-COV: a comparative overview. Infez. Med. 28, 174–184. 32275259

[26] Enjuanes L, Almazan F, Sola L, Zuniga, S 2006 Biochemical aspects of coronavirus replication and virus–host interaction. Annu. Rev. Microbiol. 60, 211–230. (10.1146/annurev.micro.60.080805.142157) 16712436

[27] Neuman BW et al 2011 A structural analysis of M protein in coronavirus assembly and morphology. J. Struct. Biol. 174, 11–22. (10.1016/j.jsb.2010.11.021) 21130884 PMC4486061

[28] Nieto-Torres JL et al. 2014 Severe acute respiratory syndrome coronavirus envelope protein ion channel activity promotes virus fitness and pathogenesis. PLoS Pathog. 10, e1004077 (10.1371/journal.ppat.1004077) 24788150 PMC4006877

[29] Bosch BJ, van der Zee R, de Haan CA, Rottier PJM. 2003 The coronavirus spike protein is a class I virus fusion protein: structural and functional characterization of the fusion core complex. J. Virol. 77, 8801–8811. (10.1128/JVI.77.16.8801-8811.2003) 12885899 PMC167208

[30] Collins AR, Knobler RL, Powell H, Buchmeier MJ 1982 Monoclonal antibodies to murine hepatitis virus-4 (strain JHM) define the viral glycoprotein responsible for attachment and cell–cell fusion. Virology 119, 358–371. (10.1016/0042-6822(82)90095-2) 6281979 PMC7130542

[31] Hoffmann M et al 2020 SARS-CoV-2 cell entry depends on ACE2 and TMPRSS2 and is blocked by a clinically proven protease inhibitor. Cell 181, 271–280. (10.1016/j.cell.2020.02.052) 32142651 PMC7102627

[32] Cornelissen LA, Wierda CM, van der Meer FJ, Herrewegh AA, Horzinek MC, Egberink HF, de Groot RJ. 1997 Hemagglutinin-esterase, a novel structural protein of torovirus. J. Virol. 71, 5277–5286. (10.1128/JVI.71.7.5277-5286.1997) 9188596 PMC191764

[33] Ong SWX, Tan YK, Chia PY, Lee TH, Ng OT, Wong MSY, Marimuthu K 2020 Air, surface environmental, and personal protective equipment contamination by severe acute respiratory syndrome coronavirus 2 (SARS-CoV-2) from a symptomatic patient. JAMA. 323, 1610–1612. (10.1001/jama.2020.3227) 32129805 PMC7057172

[34] Duan SM et al. 2003 Stability of SARS coronavirus in human specimens and environment and its sensitivity to heating and UV irradiation. Biomed. Environ. Sci. 16, 246–255. 14631830

[35] Lai MYY, Cheng PKC, Lim WWL 2005 Survival of severe acute respiratory syndrome coronavirus. Clin. Infect. Dis. 41, 67–71. (10.1086/433186) 16142653 PMC7107832

[36] Chan KH, Peiris JS, Lam SY, Poon LL, Yuen KY, Seto WH 2011 The effects of temperature and relative humidity on the viability of the SARS coronavirus. Adv. Virol. 734690 (10.1155/2011/734690) 22312351 PMC3265313

[37] Sizun J, Yu MW, Talbot PJ 2000 Survival of human coronaviruses 229E and OC43 in suspension and after drying on surfaces: a possible source of hospital-acquired infections. J. Hosp. Infect. 46, 55–60. (10.1053/jhin.2000.0795) 11023724 PMC7134510

[38] Warnes SL, Little ZR, Keevil CW 2015 Human coronavirus 229E remains infectious on common touch surface materials. mBio 6, e01697–15 (10.1128/mBio.01697-15) 26556276 PMC4659470

[39] van Doremalen N, Bushmaker T, Munster VJ. 2013 Stability of Middle East respiratory syndrome coronavirus (MERS-CoV) under different environmental conditions. Euro. Surveill. 18, 20590 (10.2807/1560-7917.es2013.18.38.20590) 24084338

[40] Chin AWH, Chu JTS, Perera MRA, Hui KPY, Yen HL, Chan MCW, Peiris M, Poon LLM 2020 Stability of SARS-CoV-2 in different environmental conditions. Microbe 1, 10 (10.1016/S2666-5247(20)30003-3) PMC7214863 32835322

[41] Santarpia JL et al 2020 Aerosol and surface contamination of SARS-CoV-2 observed in quarantine and isolation care. Sci. Rep. 10, 12732 (10.1038/s41598-020-69286-3) 32728118 PMC7391640

[42] Ren SY, Wang WB, Hao YG, Zhang HR, Wang ZC, Chen YE, Gao RD 2020 Stability and infectivity of coronaviruses in inanimate environments. World J. Clin. Cases 8, 1391–1399. (10.12998/wjcc.v8.i8.1391) 32368532 PMC7190947

[43] Gerba CP 1984 Applied and theoretical aspects of virus adsorption to surfaces. Adv. Appl. Microbiol. 30, 133–168. (10.1016/S0065-2164(08)70054-6) 6099689

[44] Casanova LM, Jeon S, Rutala WA, Weber DJ, Sobsey MD 2010 Effects of air temperature and relative humidity on coronavirus survival on surfaces. Appl. Environ. Microbiol. 76, 2712–2717. (10.1128/aem.02291-09) 20228108 PMC2863430

[45] Jones L, Walsh K, Willcox M, Morgan P, Nichols J 2020 The COVID-19 pandemic: important considerations for contact lens practitioners. Cont. Lens. Anterior. Eye 43, 196–203. (10.1016/j.clae.2020.03.012) 32273245 PMC7129028

[46] Vasickova P, Pavlik I, Verani M, Carducci A 2010 Issues concerning survival of viruses on surfaces. Food Environ. Virol. 2, 24–34. (10.1007/s12560-010-9025-6)

[47] Boone SA, Gerba CP 2007 Significance of fomites in the spread of respiratory and enteric viral disease. Appl. Environ. Microbiol. 73, 1687–1696. (10.1128/AEM.02051-06) 17220247 PMC1828811

[48] Chin AWH, Chu, JTS, Perera MRA, Hui KPY, Yen HL, Chan MCW, Peiris M, Poon LLM 2020 Stability of SARS-CoV-2 in different environmental conditions. Lancet Microbe. 1, 10 (10.1016/S2666-5247(20)30003-3) PMC7214863 32835322

[49] Castaño N, Cordts S, Jalil MK, Zhang K, Koppaka S, Bick A, Paul R, Tang SKY 2020 Fomite transmission and disinfection strategies for SARS-CoV-2 and related viruses. *arXiv*, arXiv2005, 11443.

[50] Fuhs GW, Chen M, Sturman LS, Moore RS 1985 Virus adsorption to mineral surfaces is reduced by microbial overgrowth and organic coatings. Microb. Ecol. 11, 25–39. (10.1007/BF02015106) 24221237

[51] Siddiquie RY, Agrawal A, Joshi SS 2020 Surface alterations to impart antiviral properties to combat COVID-19. Trans. Indian Natl Acad. Eng*.* 5, 343–347. (10.1007/s41403-020-00096-9) PMC7223978 38624346

[52] Armanious A, Aeppli M, Jacak R, Refardt D, Sigstam T, Kohn T, Sander M 2016 Viruses at solid–water interfaces: a systematic assessment of interactions driving adsorption. Environ. Sci. Technol. 50, 732–743. (10.1021/acs.est.5b04644) 26636722

[53] Ratnesar-Shumate S et al 2020 Simulated sunlight rapidly inactivates SARS-CoV-2 on surfaces. J. Infect. Dis. 222, 214–222. (10.1093/infdis/jiaa274) 32432672 PMC7313905

[54] Tiwari A, Patnayak DP, Chander Y, Parsad M, Goyal SM 2006 Survival of two avian respiratory viruses on porous and nonporous surfaces. Avian Dis. 50, 284–287. (10.1637/7453-101205R.1) 16863083

[55] Shim J, Stewart DS, Nikolov AD, Wasan DT, Wang R, Yan R, Shieh YC 2017 Differential MS2 interaction with food contact surfaces determined by atomic force microscopy and virus recovery. Appl. Environ. Microbiol. 83, e01881–17 (10.1128/AEM.01881-17) PMC5717206 28986376

[56] Dika C, Ly-Chatain MH, Francius G, Duval JFL, Gantzer C 2013 Non-DLVO adhesion of F-specific RNA bacteriophages to abiotic surfaces: importance of surface roughness, hydrophobic and electrostatic interactions. Colloids. Surf. A. Physicochem. Eng. Asp. 435, 178–187. (10.1016/j.colsurfa.2013.02.045)

[57] Rabenau HF, Cinatl J, Morgenstern B, Bauer G, Preiser W, Doerr HW 2005 Stability and inactivation of SARS coronavirus. Med. Microbiol. Immunol. 194, 1–6. (10.1007/s00430-004-0219-0) 15118911 PMC7086689

[58] Siddharta A et al 2017 Virucidal Activity of World Health Organization-recommended formulations against enveloped viruses, Including Zika, Ebola, and emerging coronaviruses. J. Infect. Dis. 215, 902 (10.1093/infdis/jix046) 28453839 PMC5407053

[59] Rabenau HF, Kampf G, Cinatl J, Doerr HW 2017 Efficacy of various disinfectants against SARS coronavirus. J. Hosp. Infect. 61, 107–111. (10.1016/j.jhin.2004.12.023) PMC7132504 15923059

[60] Eggers M, Eickmann M, Zorn J 2015 Rapid and effective virucidal activity of povidone-iodine products against Middle East Respiratory Syndrome Coronavirus (MERS-CoV) and Modified VacciniaVirus Ankara (MVA). Infect. Dis. Ther. 4, 491–501. (10.1007/s40121-015-0091-9) 26416214 PMC4675768

[61] Eggers M, Koburger-Janssen T, Eickmann M, Zorn J 2018 *In vitro* bactericidal and virucidal efficacy of povidone-iodine gargle/mouthwash against respiratory and oral tract pathogens. Infect. Dis. Ther. 7, 249–259. (10.1007/s40121-018-0200-7) 29633177 PMC5986684

[62] Omidbakhsh N, Sattar SA 2006 Broad-spectrum microbicidal activity, toxicologic assessment, and materials compatibility of a new generation of accelerated hydrogen peroxide-based environmental surface disinfectant. Am. J. Infect. Control. 34, 251–257. (10.1016/j.ajic.2005.06.002) 16765201 PMC7132737

[63] Wood A, Payne D 1998 The action of three antiseptics/disinfectantsagainst enveloped and non-enveloped viruses. J. Hosp. Infect. 38, 283–295. (10.1016/s0195-6701(98)90077-9) 9602977 PMC7134397

[64] AFNOR. 2007 Chemical disinfectants and— Virucidal quantitative suspension test for chemical disinfectants and antiseptics used in human medicine—Test method and requirements (phase 2, step 1). NF EN 14476+A1.

[65] WHO. 2014 Use of disinfectants: alcohol and bleach. In Infection prevention and control of epidemic-and pandemic-prone acute respiratory infections in health care, pp. 65–66. Geneva, Switzerland: WHO. 24983124

[66] Sattar SA, Springthorpe VS, Karim Y, Loro P 1989 Chemical disinfection of non-porous inanimate surfaces experimentally contaminated with four human pathogenic viruses. Epidemiol. Infect. 102, 493–505. (10.1017/s0950268800030211) 2737256 PMC2249473

[67] Rutala WA, Peacock JE, Gergen MF, Sobsey MD, Weber DJ 2006 Efficacy of hospital germicides against adenovirus 8, a common cause of epidemic keratoconjunctivitis in health care facilities. Antimicrob. Agents Chemother. 50, 1419–1424. (10.1128/AAC.50.4.1419-1424.2006) 16569860 PMC1426955

[68] Milovanovic M, Arsenijevic A, Milovanovic J, Kanjevac T, Arsenijevic N 2017 Nanoparticles in antiviral therapy. In Antimicrobial nanoarchitectonics: from synthesis to applications, pp. 383–410. Amsterdam, Netherlands: Elsevier (10.1016/B978-0-323-52733-0.00014-8)

[69] Talebain S, Wallace GC, Schroeder A, Stellacci F, Conde J 2020 Nanotechnology-based disinfectants and sensors for SARS-CoV-2. Nat. Nanotechnol. 15, 618–621. (10.1038/s41565-020-0751-0) 32728083

[70] Hahn A, Fuhlrott J, Loos A, Barcikowski, S 2012 Cytotoxicity and ion release of alloy nanoparticles. J. Nanopart. Res. 14, 1–10. (10.1007/s11051-011-0686-3) 22448125 PMC3309564

[71] te Velthuis AJW, van den Worm SHE, Sims AC, Baric RS, Snijder EJ, van Hemert MJ 2010 Zn^2+^ inhibits coronavirus and arterivirus RNA polymerase activity *in vitro* and zinc ionophores block the replication of these viruses in cell culture. PLoS Pathog. 6, e1001176 (10.1371/journal.ppat.1001176) 21079686 PMC2973827

[72] Jeong Y, Lim DW, Choi J 2014 Assessment of size-dependent antimicrobial and cytotoxic properties of silver nanoparticles. Adv. Mater. Sci .Eng. 2014, 1–6. (10.1155/2014/763807)

[73] Yu Z, Li Q, Wang J, Yu Y, Wang Y, Zhou Q, Li P 2020 Reactive oxygen species-related nanoparticle toxicity in the biomedical field. Nanoscale Res. Lett. 15, 115 (10.1186/s11671-020-03344-7) 32436107 PMC7239959

[74] Kerry RG, Malik S, Redda YT, Sahoo S, Patra JK, Majhi S 2019 Nano-based approach to combat emerging viral (NIPAH virus) infection. Nanomedicine 18, 196–220. (10.1016/j.nano.2019.03.004) 30904587 PMC7106268

[75] Di Gianvincenzo P, Marradi M, Martínez-Avila OM, Bedoya LM, Alcamí J, Penadés S. 2010 Gold nanoparticles capped with sulfate-ended ligands as anti-HIV agents. Bioorg. Med. Chem. Lett. 20, 2718–2721. (10.1016/j.bmcl.2010.03.079) 20382017

[76] Elechiguerra JL, Burt JL, Monroes JR, Camacho-Bragado A, Gao X, Lara HH, Yacaman MJ 2005 Interaction of silver nanoparticles with HIV-1. J. Nanobiotechnol. 3, 6 (10.1186/1477-3155-3-6) PMC1190212 15987516

[77] Sportelli MC, Izzi M, Kukushkina EA, Hossain SI, Picca RA, Ditaranto N, Cioffi N 2020 Can nanotechnology and materials science help the fight against SARS-CoV-2. Nanomaterials 10, 802 (10.3390/nano10040802) PMC7221591 32326343

[78] Kampf G, Voss A, Scheihauer S 2020 Inactivation of coronaviruses by heat. J. Hosp. Infect. 105, 348–349. (10.1016/j.jhin.2020.03.025) 32243951 PMC7271332

[79] Weiss C et al 2020 Toward nanotechnology-enabled approaches against the COVID-19 pandemic. ACS Nano 14, 6383–6406. (10.1021/acsnano.0c03697) 32519842

[80] Nazari M et al. 2017 Plasmonic enhancement of selective photonic virus inactivation. Sci. Rep. 7, 11951 (10.1038/s41598-017-12377-5) 28931903 PMC5607298

[81] Nakano R, Ishiguro H, Yao Y, Kajioka J, Fujishima A, Sunada K, Minoshima M, Hashimoto K, Kubota Y 2012 Photocatalytic inactivation of influenza virus by titanium dioxide thin film. Photochem. Photobiol. Sci. 11, 1293–1298. (10.1039/C2PP05414K) 22580561

[82] Matharu RK, Porwal H, Chen B, Ciric L, Edirisinghe M 2020 Viral filtration using carbon-based materials. Med. Devices Sens. 3, e10107 (10.1002/mds3.10107) PMC7323107 32838209

[83] Matharu RK, Ciric L, Ren G, Edirisinghe M 2020 Comparative study of the antimicrobial effects of tungsten nanoparticles and tungsten nanocomposite fibres on hospital acquired bacterial and viral pathogens. Nanomaterials 10, 1017 (10.3390/nano10061017) PMC7352352 32466574

[84] Srivastava AK, Dwivedi N, Dhand C, Khan R, Sathish N, Gupta, MK, Kumar R, Kumar S 2020 Potential of graphene-based materials to combat COVID-19: properties, perspectives, and prospects. Mater. Today Chem. 18, 100385 (10.1016/j.mtchem.2020.100385) 33106780 PMC7577689

[85] Raghav PK, Mohanty S 2020 Are graphene and graphene-derived products capable of preventing COVID-19 infection? Med. Hypotheses. 144, 110031 (10.1016/j.mehy.2020.110031) 33254479 PMC7313523

[86] Zhou Y, Hou Y, Shen J, Huang Y, Martin W, Cheng F 2020 Network-based drug repurposing for novel coronavirus 2019-nCoV/SARS-CoV-2. Cell Discov. 6, 14 (10.1038/s41421-020-0153-3) 32194980 PMC7073332

[87] Sheahan TP et al. 2020 Comparative therapeutic efficacy of remdesivir and combination lopinavir, ritonavir, and interferon beta against MERS-CoV. Nat. Commun. 11, 222 (10.1038/s41467-019-13940-6) 31924756 PMC6954302

[88] Dyall J, Gross R, Kindrachuk J, Johnson RF, Olinger GG Jr, Hensley LE, Frieman MB, Jahrling PB 2017 Middle East respiratory syndrome and severe acute respiratory syndrome: current therapeutic options and potential targets for novel therapies. Drugs 77, 1935–1966. (10.1007/s40265-017-0830-1) 29143192 PMC5733787

[89] McKee DL, Sternberg A, Stange U, Laufer S, Naujokat C 2020 Candidate drugs against SARS-CoV-2 and COVID-19. Pharmacol. Res. 157, 04859 (10.1016/j.phrs.2020.104859) PMC7189851 32360480

[90] Fehr AR, Perlman S 2015 Coronaviruses: an overview of their replication and pathogenesis. Methods Mol. Biol. 1282, 1–23. (10.1007/978-1-4939-2438-7_1) 25720466 PMC4369385

[91] Zhou Y, Simmons G 2012 Development of novel entry inhibitors targeting emerging viruses. Expert. Rev. Anti. Infect. Ther. 10, 1129–1138. (10.1586/eri.12.104) 23199399 PMC3587779

[92] Zhou Y et al. 2015 Protease inhibitors targeting coronavirus and filovirus entry. Antiviral Res. 116, 76–84. (10.1016/j.antiviral.2015.01.011) 25666761 PMC4774534

[93] Martin WR, Cheng F 2020 Repurposing of FDA-approved toremifene to treat COVID-19 by blocking the spike glycoprotein and NSP14 of SARS-CoV-2. ChemRxiv . (10.26434/chemrxiv.12431966.v1)

[94] Ho TY, Wu SL, Chen JC, Li CC, Hsiang CY 2007 Emodin blocks the SARS coronavirus spike protein and angiotensin-converting enzyme 2 interaction. Antiviral Res. 74, 92–101. (10.1016/j.antiviral.2006.04.014) 16730806 PMC7114332

[95] Caly L, Druve JD, Catton MG, Jans DA, Wagstaff KM 2020 The FDA-approved drug ivermectin inhibits the replication of SARS-CoV-2 *in vitro*. Antiviral Res. 178, 104787 (10.1016/j.antiviral.2020.104787) 32251768 PMC7129059

[96] Fantini J, Scala CD, Chahinian H, Yahi N 2020 Structural and molecular modelling studies reveal a new mechanism of action of chloroquine and hydroxychloroquine against SARS-CoV-2 infection. Int. J. Antimicrob. Agents. 55, 105960 (10.1016/j.ijantimicag.2020.105960) 32251731 PMC7128678

[97] Chung YH, Beiss V, Fiering SN, Steinmetz NF 2020 COVID-19 vaccine frontrunners and their nanotechnology design. ACS Nano 14, 12 522–12 537. (10.1021/acsnano.0c07197) 33034449

[98] Campos EVR, Pereira AES, de Oliveira JL, Carvalho LB, Guilger-Casagrande M, de Lima R, Fraceto LF. 2020 How can nanotechnology help to combat COVID-19? Opportunities and urgent need. J. Nanobiotechnol. 18, 125–147. (10.1186/s12951-020-00685-4) PMC7474329 32891146

[99] Udugama B et al. 2020 Diagnosing COVID-19: the disease and tools for detection. ACS Nano 14, 3822–3835. (10.1021/acsnano.0c02624) 32223179

[100] Tabish TA, Narayan RJ, Edirisinghe M 2020 Rapid and label-free detection of COVID-19 using coherent anti-Stokes Raman scattering microscopy. MRS Commun. 10, 566–572. (10.1557/mrc.2020.81) 33398237 PMC7773019

[101] Yan Y, Chang L, Wang L 2020 Laboratory testing of SARS-CoV, MERS-CoV, and SARS-CoV-2 (2019-nCoV): current status, challenges, and countermeasures. Rev. Med. Virol. 30, e2106 (10.1002/rmv.2106) 32302058 PMC7235496

[102] Zhu X (2020).

[103] Chen HW, Fang ZS, Chen YT, ChenYI, Yao BY, Cheng JY, Chien CY, Chang YC, Hu CMJ 2017 Targeting and enrichment of viral pathogen by cell membrane cloaked magnetic nanoparticles for enhanced detection. ACS Appl. Mater. Interfaces 9, 39 953–39 961. (10.1021/acsami.7b09931) 29088538

[104] Medhi R, Srinoi P, Ngo N, Tran HV, Lee TR 2020 Nanoparticle-based strategies to combat COVID-19. ACS Appl. Nano Mater. 3, 8557–8580. (10.1021/acsanm.0c01978) 37556239

[105] Rao L et al 2020 Decoy nanoparticles protect against COVID-19 by concurrently adsorbing viruses and inflammatory cytokines. Proc. Natl Acad. Sci. USA 117, 27 141–27 147. (10.1073/pnas.2014352117) PMC7959535 33024017

[106] Paliwal P, Sargolzaei S, Bhardwaj SK, Bhardwaj V, Dixit C, Kaushik AG 2020 Challenges in bio-nanotechnology to manage the COVID-19 pandemic. Front. Nanotechnol. 2, 2673–3013. (10.3389/fnano.2020.571284)

[107] Bhavana V, Thakor P, Singh SB, Mehra, NK 2020 COVID-19: pathophysiology, treatment options, nanotechnology approaches, and research agenda to combating the SARS-CoV2 pandemic. Life Sci. 261, 118336 (10.1016/j.lfs.2020.118336) 32846164 PMC7443335

[108] Talebian S, Conde J 2020 Why go NANO on COVID-19 pandemic? Matter 3, 598–601. (10.1016/j.matt.2020.08.005) 32905308 PMC7466941

[109] Florindo HF, Kleiner R, Vaskovich-Koubi D, Acúrcio RC, Carreria B, Yeini E, Tiram G, Liubomirski S-FR 2020 Immune-mediated approaches against COVID-19. Nat. Nanotechnol. 15, 630–645. (10.1038/s41565-020-0732-3) 32661375 PMC7355525

[110] Shin MD et al. 2020 COVID-19 vaccine development and a potential nanomaterial path forward. Nat. Nanotechnol. 15, 646–655. (10.1038/s41565-020-0737-y) 32669664

